# Supplementary material for: Structural basis of ALC1/CHD1L autoinhibition and the mechanism of activation by the nucleosome
Source: Nat Commun. 2021 Jul 1;12:4057. doi: 10.1038/s41467-021-24320-4 (PMC8249414; doi:10.1038/s41467-021-24320-4)
Supplement: Supplementary file 1 — Supplementary Information [file 41467_2021_24320_MOESM1_ESM.pdf]

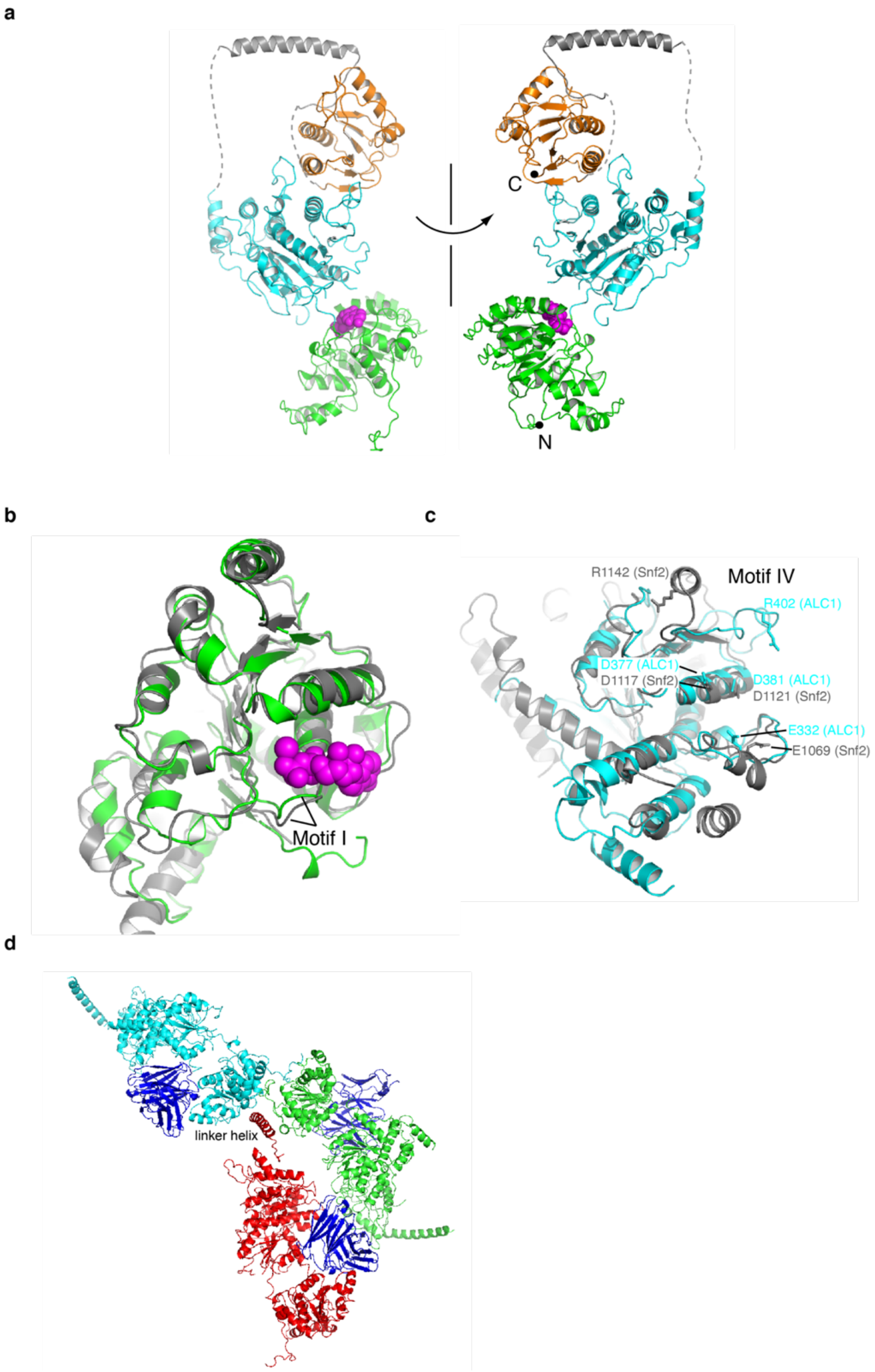

Supplementary Figure 1 | Additional analysis of the crystal structure of ALC1

(**a**) Two different views of the structure of ALC1 (colored coded) with the antibody scFv omitted for clarity. (**b-c**) Structural comparison of ALC1 (colored coded) and Snf2 (colored grey, PDB 5Z3U)[1] at lobe 1 (b) and lobe 2 (c). (**d**) The linker helix of ALC1 is involved in packing to the nearby molecules in the crystals. Three copies of ALC1 (colored red, green and cyan, respectively) in the crystal lattice are shown.

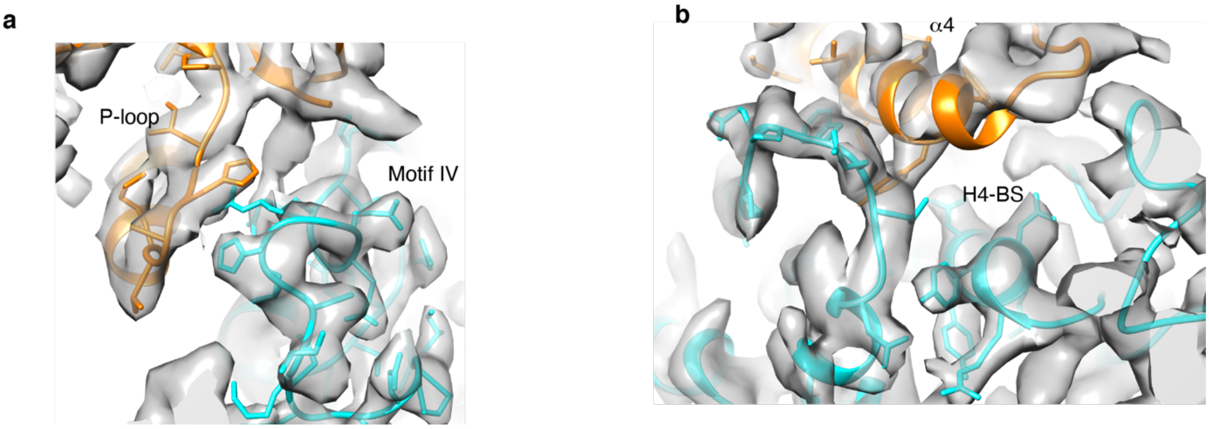

Supplementary Figure 2 | 2FoFc electron density maps of the crystal structure of ALC1

(**a**) At the interface between the macro domain and motif IV. (**b**) At the interface between the macro domain and the H4-binding surface (H4-BS). The figure was generated with Chimera[2] and the map density was shown at a contour level of σ=1.

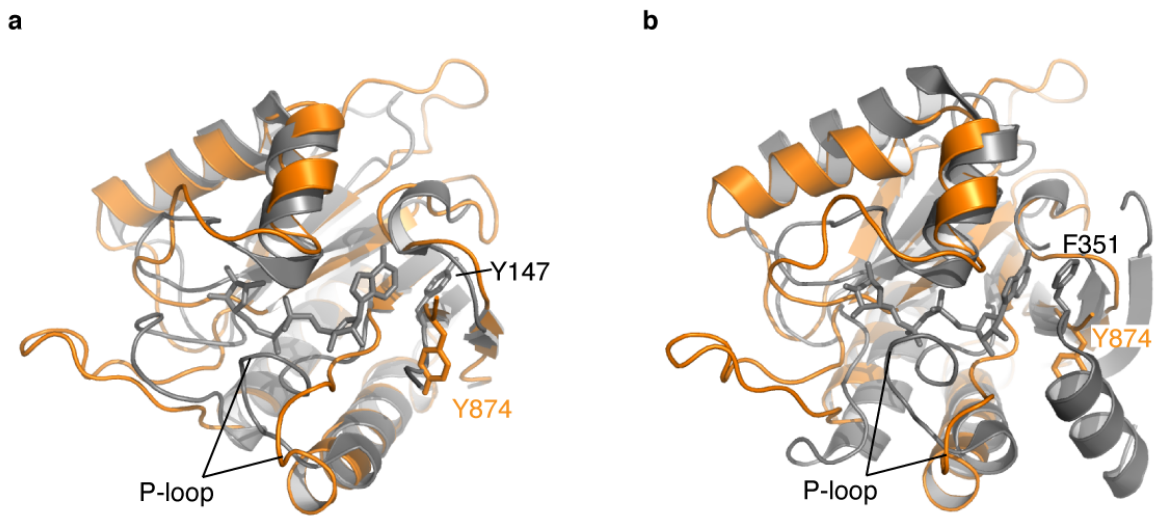

Supplementary Figure 3 | Structural comparison of the macro domains

(**a**) Alignment of the ALC1 macro domain (orange) to that of DarG (colored grey, PDB code 5m3e)[3]. (**b**) Alignment to the macro domain of macroH2A1.1 (colored grey, PDB code 3iid)[4].

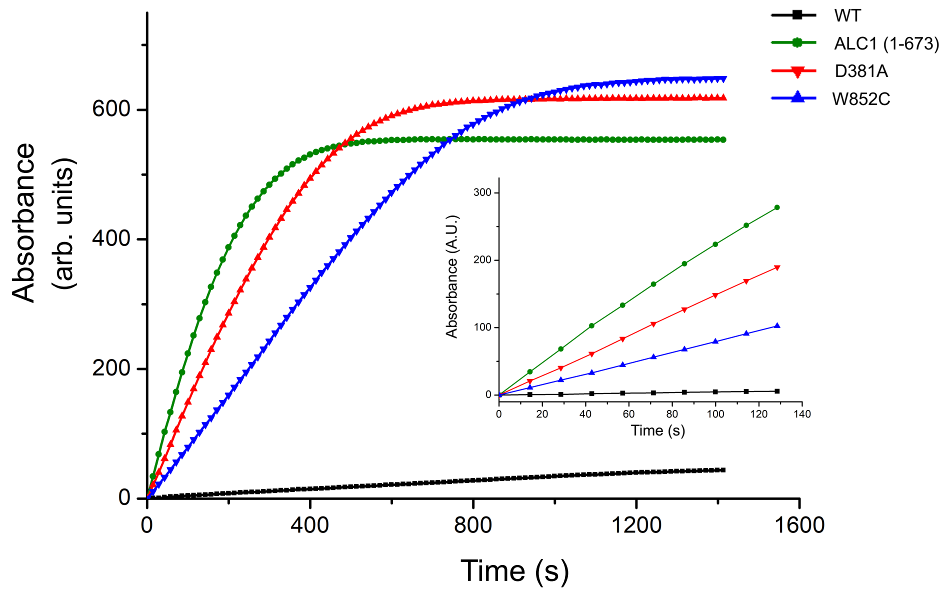

Supplementary Figure 4 | Measurement of the DNA-dependent ATPase activities of WT and three auto-inhibition mutant ALC1

In the MESG-based ATPase assays, 3 mM ATP, which is in excess over the enzyme (0.1 μM), were used. The reactions led to substrate depletion at the very end of the reactions. So, the early time points, the linear fractions of the reactions, were used to compare the activities of varied proteins. Under these conditions, the ATP substrate was not depleted before the time point of 120s.

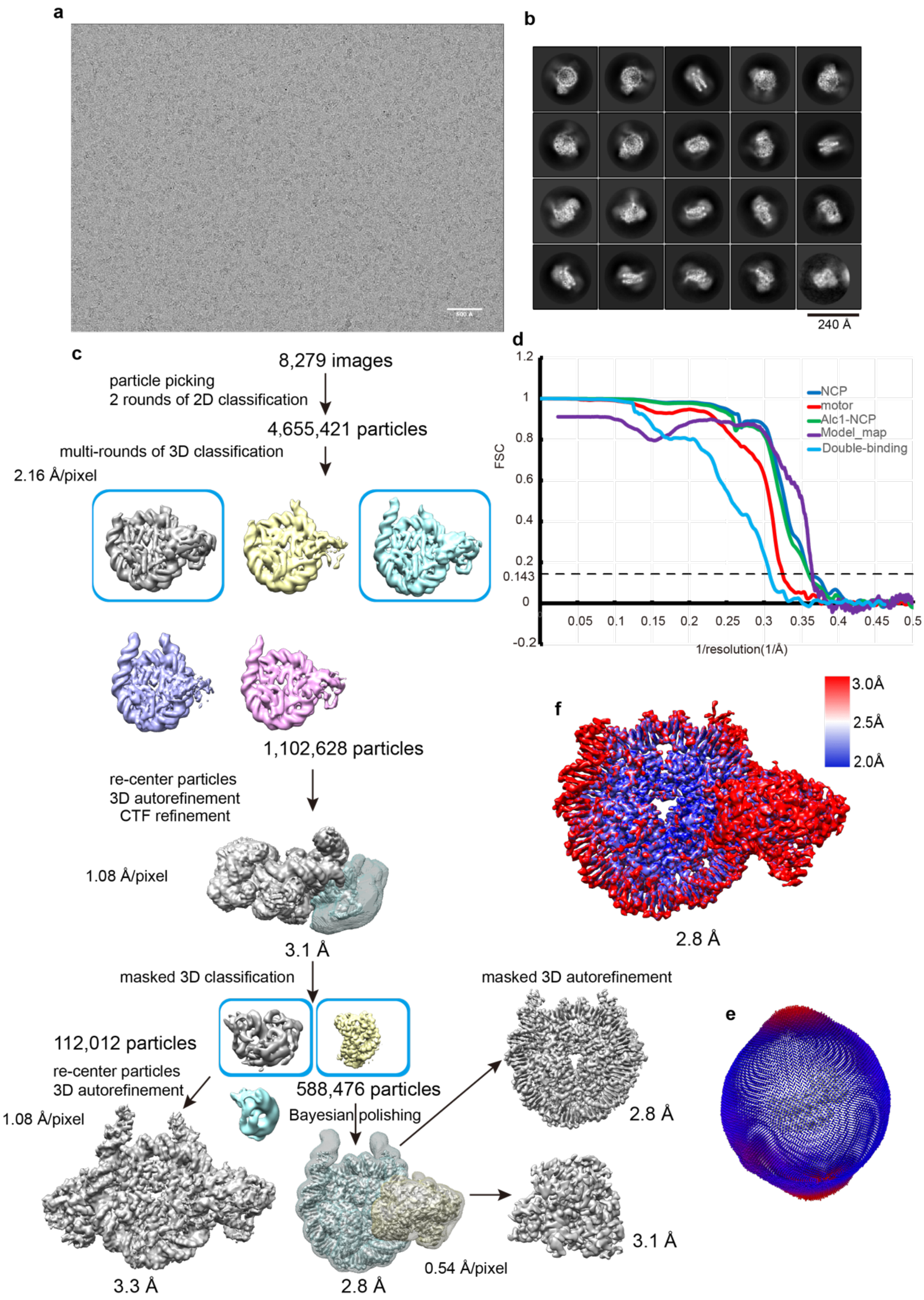

Supplementary Figure 5 | CryoEM analysis of the ALC1-nucleosome complex.

(**a**) A representative cryo-EM micrograph.

(**b**) 2D class averages of characteristic projection views of cryo-EM particles.

(**c**) Flowchart of the cryo-EM data processing for the ALC1-nucleosome dataset.

(**d**) Resolution estimation of the EM maps. Gold standard Fourier shell correlation (FSC) curves, showing the overall nominal resolutions of 2.8 Å, 3.1 Å, 2.8 Å and 3.3 Å for the whole complex, the motor region, and the nucleosome region bound with the ALC1 linker, and the double-binding complex, respectively.

(**e**) Angular distributions of the cryo-EM particles in the final round of refinement of the ALC1-NCP complex.

(**f**) Local resolution estimation of the EM map of the ALC1-nucleosome complex.

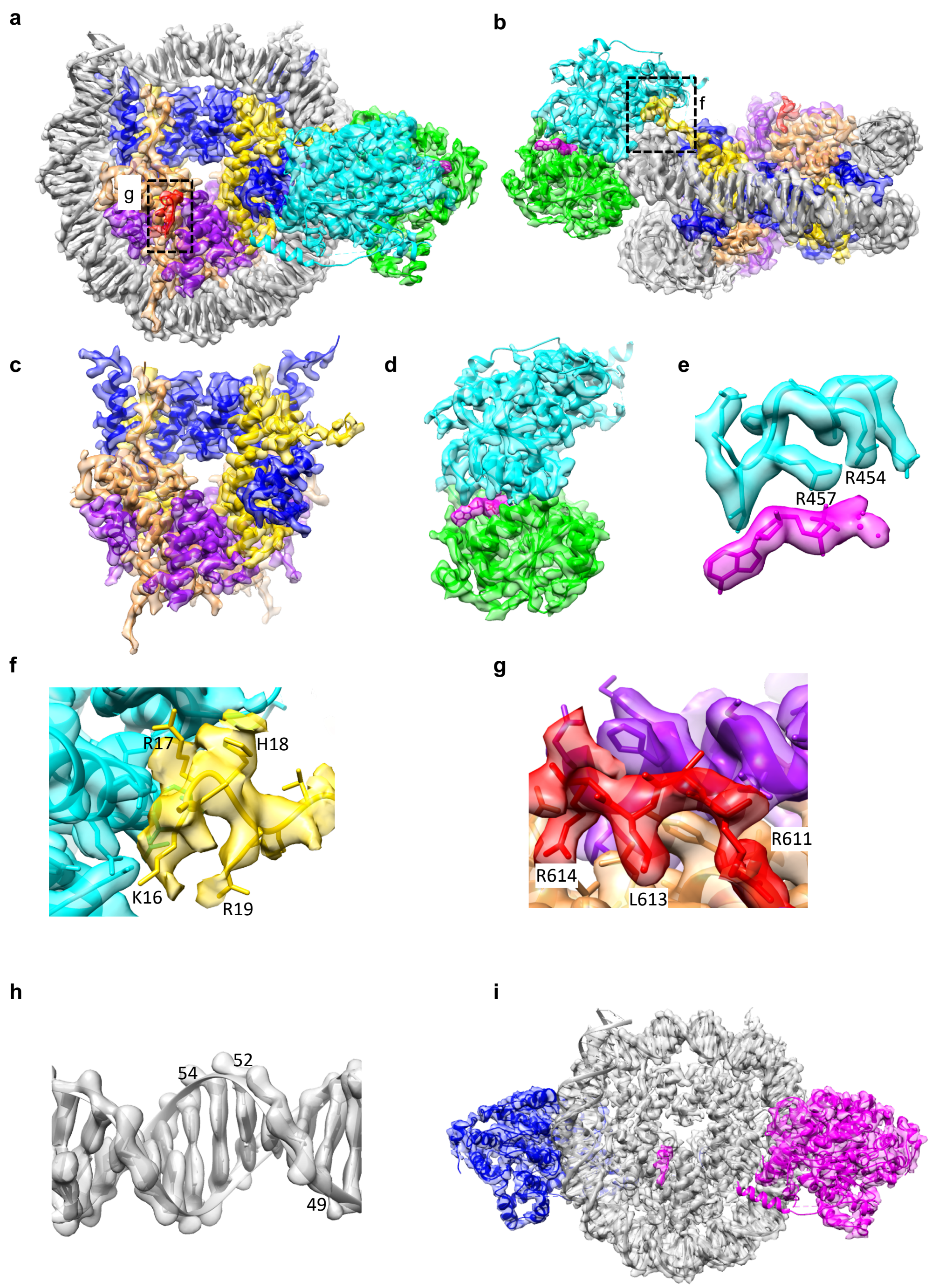

Supplementary Figure 6 | EM density maps of the ALC1-nucleosome complex (**a-b**) Two views of the overall map superimposed with the model. The encircled regions are enlarged for further analysis in (f) and (g). (**c**) Map of the histone octamer. (**d**) Map of the motor domain. (**e**) Map of the ATPase active site. (**f**) Map around the H4 tail bound by ALC1. (**g**) Map around the linker bound by H2A-H2B. (**h**) Map of the DNA at SHL2. (**i**) Map of the double-binding mode.

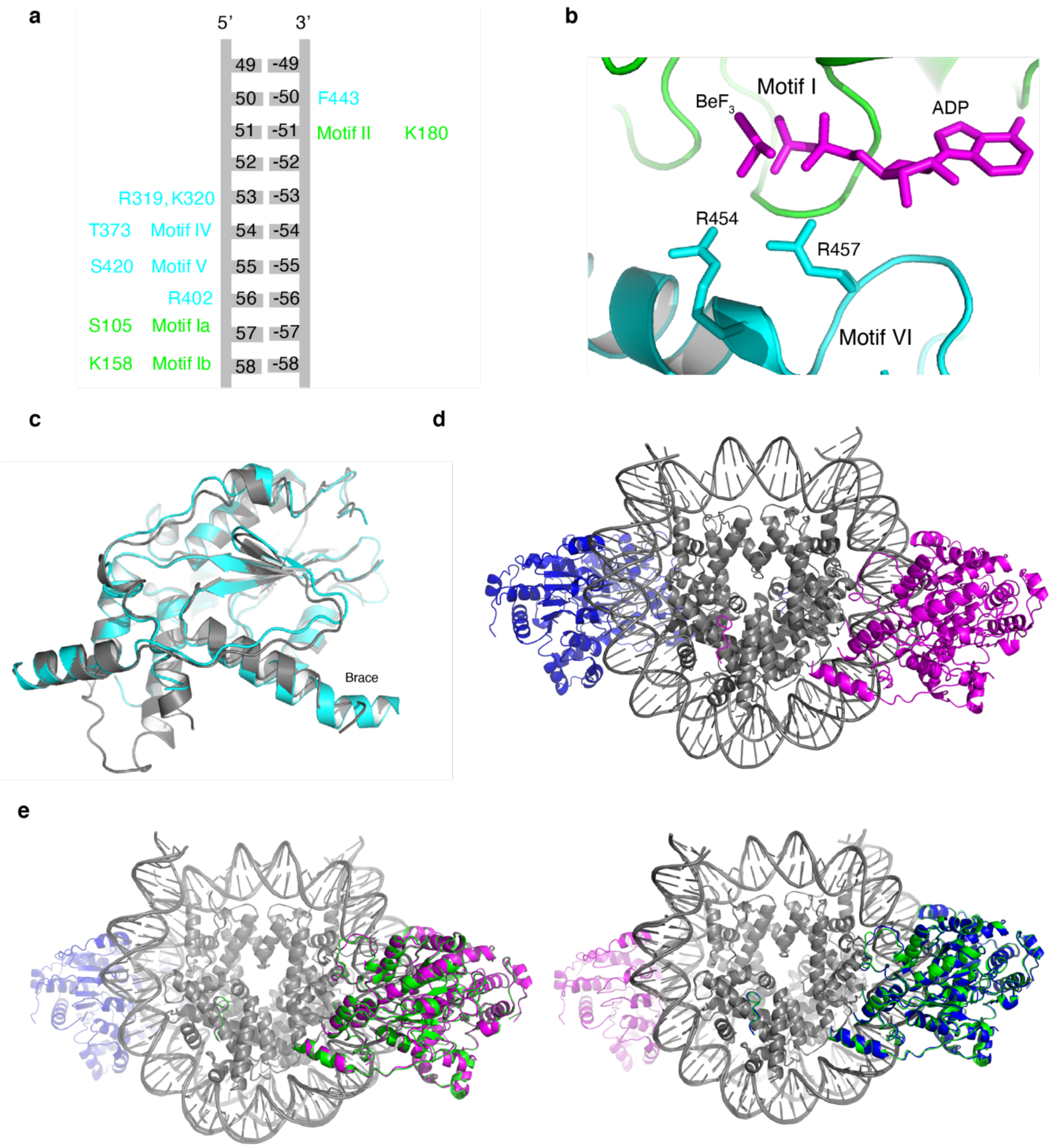

Supplementary Figure 7 | The activated conformation of ALC1 (**a**) Schematic of the interaction between ALC1 and the nucleosomal DNA. The residues in lobe 1 and lobe 2 are colored green and cyan, respectively. (**b**) Structure of the ADP-BeF$_x$ binding interface. (**c**) Structural comparison of lobe 2 in the nucleosome-bound, activated (cyan) and nucleosome-free, autoinhibited (grey) states. (**d**) Structure of the complex with double-binding ALC1. (**e**) Structural comparison of the single binding ALC1 (colored green) and the double-binding ALC1 (colored magenta and blue, respectively). The histone cores are aligned.

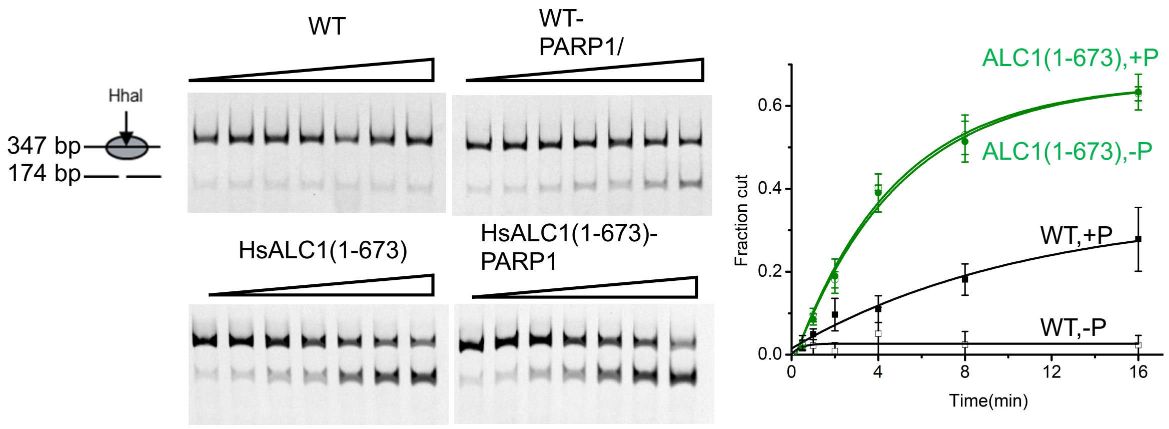

Supplementary Figure 8 | Chromatin remodeling activities of WT and ALC1 (1-673). Representative gels of the chromatin remodeling assays are shown. Quantification of the activities is shown on the right. Black, WT; green, ALC1 (1-673). Open squares, activities measured without PARP1 (-P); solid squares, activities measured in the presence of PARP1 (+P). A low concentration (0.04 µM) of WT and ALC1 (1-673) proteins were used due to the high activity of ALC1 (1-673). Error bars indicate standard deviations for three independent measurements and the measure of centre for the error bars is the mean value.

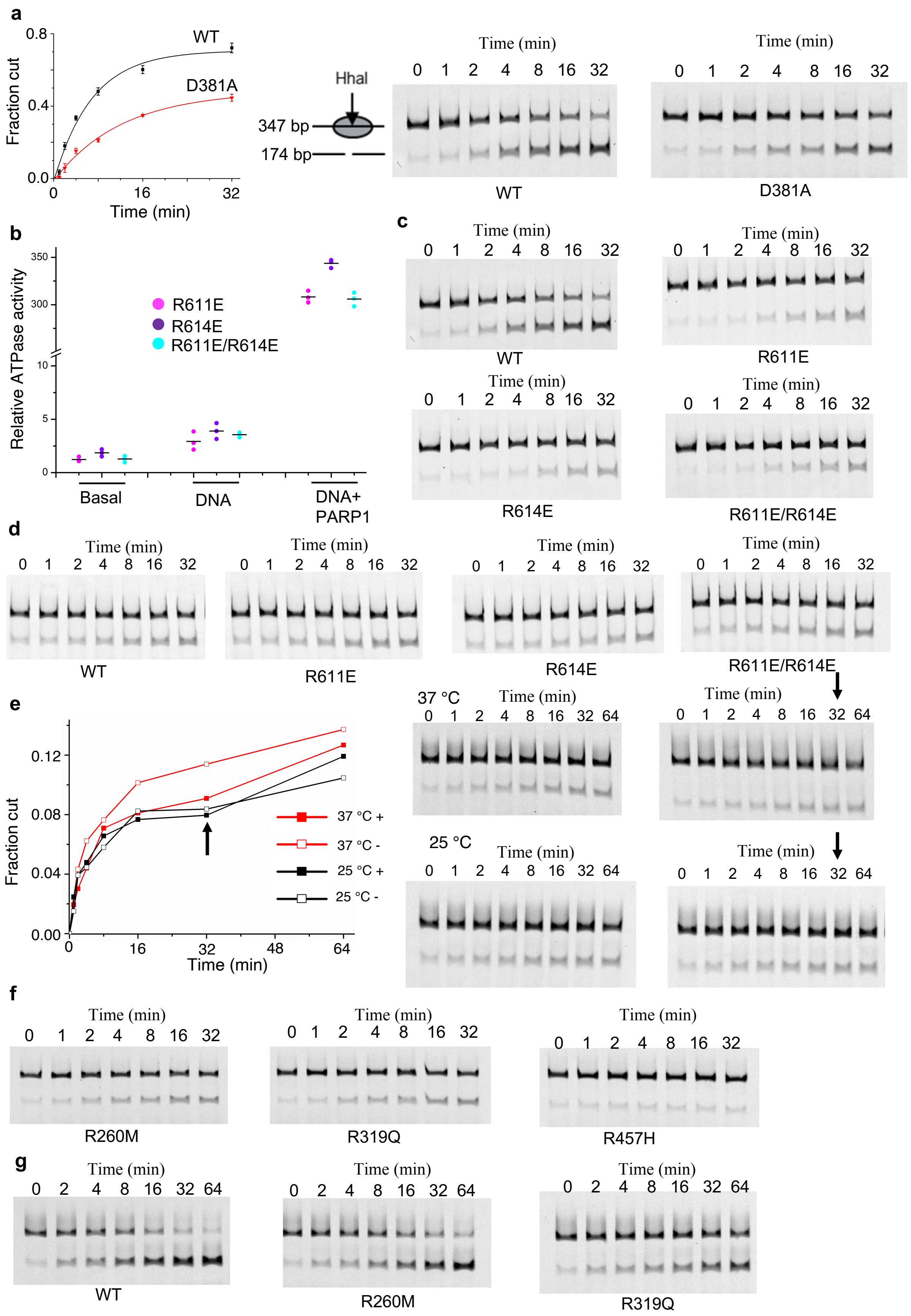
a
Fraction cut
Time (min)
WT
D381A
HhaI
347 bp
174 bp
Time (min)
0 1 2 4 8 16 32
WT
D381A
b
Relative ATPase activity
R611E
R614E
R611E/R614E
Basal
DNA
DNA+ PARP1
c
Time (min)
WT
R611E
R614E
R611E/R614E
d
Time (min)
WT
R611E
R614E
R611E/R614E
e
Fraction cut
Time (min)
37 °C +
37 °C -
25 °C +
25 °C -
37 °C
25 °C
0 1 2 4 8 16 32 64
f
Time (min)
R260M
R319Q
R457H
g
Time (min)
0 2 4 8 16 32 64
WT
R260M
R319Q

Supplementary Figure 9 | Activities of the ALC1 mutants. (**a**) The chromatin remodeling activities of WT (100 nM) and the D381A mutant (100 nM) in the presence of PARP1. Error bars indicate standard deviations for three independent measurements and the measure of centre for the error bars is the mean value. Representative gels shown on the right. (**b**) ATPase activities of R611E, R614E, and R611E/R614E ALC1 mutants. All data points are showed with lines indicating the means (n=3). (**c-d**) Representative gels of the chromatin remodeling assays of WT, R611E, R614E and R611E/R614E mutants in the presence of PARP1 towards the intact nucleosome (c) and the AP mutant nucleosome (d). Three independent assays were performed and one was shown. Quantification of the activities is shown in Fig. 4d. (**e**) Chromatin remodeling of the R611E mutant performed at 37 °C (red) and 25 °C (black). Solid and unfilled squares, with and without more R611E added, respectively. Arrows indicate the time point (32 min) more R611E added to the reaction system. Gels of the remodeling reactions are shown on the right. (**f-g**) Representative gels of the chromatin remodeling assays of the indicated enzymes performed at 37 °C (f) and 25 °C (g). Three independent assays were performed and one was shown. Quantification of the activities is shown in Fig. 5g and 5h, respectively.

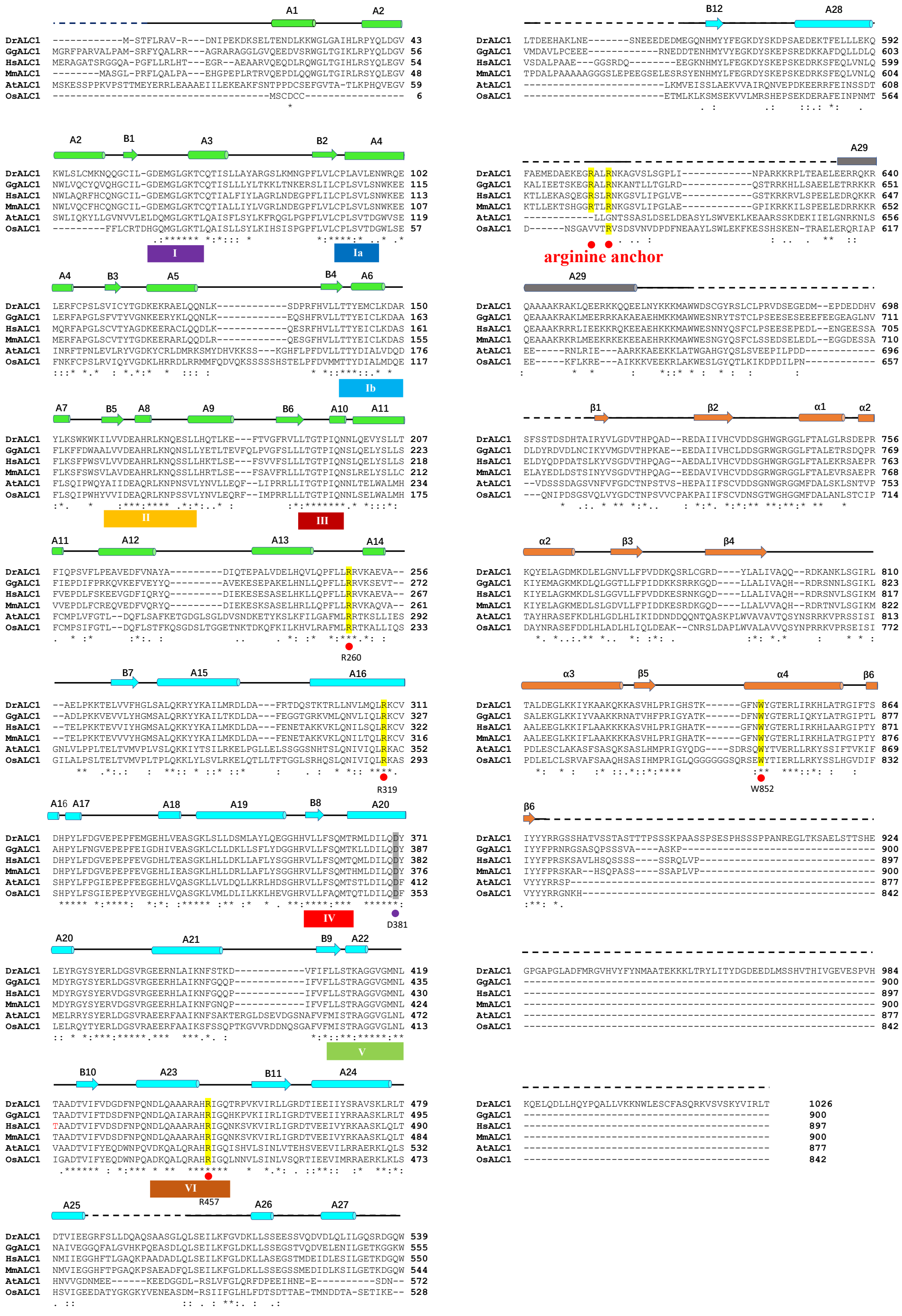

Supplementary Figure 10 | Multiple sequence alignments of ALC1 homologs. The alignments are performed with Clustal Omega Server[5]. Secondary structural assignments of the motor and macro domains are based on the cryoEM and crystal structures, respectively. The residues mutated in the study are indicated with dots, with the cancer-associated ones in red. The helicase motifs, such as motif I (P-loop), and arginine anchor are indicated.

Supplementary Table 1 Data collection and refinement statistics (molecular replacement)

**Data collection**	ALC1 (PDB code 7EPU)
Space group	$C222_1$
Cell dimensions	
a, b, c(Å)	153.480, 225.263, 106.533
α, β, γ(°)	90, 90, 90
Resolution(Å)	50.00-3.50(3.56-3.50)
Rsym or Rmerge	0.059(0.693)
I/δI	17.4(1.3)
Completeness(%)	99.4(97.5)
Redundancy	10.8(9.9)
CC1/2	0.51
**Refinement**	
Resolution(Å)	45.40-3.50
No.reflections	23428
Rwork or Rfree	26.8/31.6
No.atoms	
Protein	7787
Ligand/ion	/
Water	/
B-factors	
Protein	128
Ligand/ion	129
Water	/
R.m.s. deviations	
Bond lengths(Å)	0.004
Bond angles(°)	0.644

*Values in parentheses are for highest-resolution shell.

Supplementary. Table 2 Cryo-EM data collection, refinement and validation statistics

	#1 ALC1-NCP (EMD-31217, PDB 7ENN)	#2 NCP (EMD-31217)	#3 motor (EMD-31217)
**Data collection and processing**			
Microscope	Krios G3i	Krios G3i	Krios G3i
Camera	K3	K3	K3
Magnification (nominal)	81000	81000	81000
Electron exposure ($e^-/Å^2$)	50	50	50
Number of frames collected	32	32	32
Energy filter slit width (eV)	20	20	20
Automation software	AutoEMation2	AutoEMation2	AutoEMation2
Voltage (kV)	300	300	300
Micrographs (no.)	8279	8279	8279
Defocus range (μm)	-1.3— -1.8	-1.3— -1.8	-1.3— -1.8
Pixel size (Å)	0.54125	0.54125	0.54125
Symmetry imposed	C1	C1	C1
Initial particle images (no.)	4,655,228	4,655,228	4,655,228
Final particle images (no.)	586,673	586,673	586,673
Error of translations	1.114	1.130	2.173
Error of rotations	1.335	1.469	2.604
Map resolution (Å) (masked)	2.79	2.79	3.09
FSC threshold	0.143	0.143	0.143
Map sharpening *B* factor ($Å^2$)	-30	-30	-30
**Refinement**			
Initial model used (PDB code)	5Z3V		
Refinement package	Phenix		
Model-map scores			
CC(mask)	0.87		
CC(box)	0.84		
CC(peaks)	0.78		
CC(volume)	0.85		
R.m.s. deviations			
Bond lengths (Å)	0.005		
Bond angles (°)	0.636		
C-beta deviation	0		
EMRinger score	4.05		
CaBLAM outliers	1.36		
**Validation**			
MolProbity score	1.63		
Clashscore	5.74		
Poor rotamers (%)	0.00		
Ramachandran plot			
Favored (%)	95.45		
Allowed (%)	4.47		
Disallowed (%)	0.08		

Supplementary. Table 3 Primer list

Primer Name	Sequence
pet28b-ALC1_1-880:F	GGGTACCACGCGTGGATCCGGAGGTAGTACAATGGAGCGCGCGGGCGCTA
pet28b-ALC1_1-880:R	TGGTGCTCGAGTGCGGCCGCATTAAGACTTGCTTCTAGGAAAATA
pet28b-ALC1_1-880:F	GGGTACCACGCGTGGATCCGGAGGTAGTACAATGGAGCGCGCGGGCGCTA
pet28b-ALC1_1-880-6his:R1	TGGTGCTCGAGTGCGGCCGCATTAGTGATGATGGTGGTGATGGCCG
pet28b-ALC1_1-880-6his:R2	GATGATGGTGGTGATGGCCGCTGCTAGACTTGCTTCTAGGAAAATA
pet28b-ALC1_1-880:R857Q_F	CTGGTATGGTACTGAGCAACTTATTCGGAAACA
pet28b-ALC1_1-880:R857Q_R	TGTTTCCGAATAAGTTGCTCAGTACCATACCAG
pet28b-ALC1_1-673:F	GGGTACCACGCGTGGATCCGGAGGTAGTACAATGGAGCGCGCGGGCGCTA
pet28b-ALC1_1-673:R	TGGTGCTCGAGTGCGGCCGCATTACTTTTTCTTATGTTCAGCCTC
pet28b-ALC1_1-880:R611E_F	GAAAGCTAGTCAAGAGGGCGAATCACTCCGAAATAAAGGCAG
pet28b-ALC1_1-880:R611E_R	CTGCCTTTATTTCGGAGTGATTCGCCCTCTTGACTAGCTTTC
pet28b-ALC1_1-880:R614E_F	CAAGAGGGCCGATCACTCGAAAATAAAGGCAGTGTTCTCATC
pet28b-ALC1_1-880:R614E_R	GATGAGAACACTGCCTTTATTTTCGAGTGATCGGCCCTCTTG
pet28b-ALC1_1-880:R611/614E_F	GAAAGCTAGTCAAGAGGGCGAATCACTCGAAAATAAAGGCAGTGTTCTCATC
pet28b-ALC1_1-880:R611/614E_R	GATGAGAACACTGCCTTTATTTTCGAGTGATTCGCCCTCTTGACTAGCTTTC
pet28b-ALC1_1-880:R260M_F	CTTGCAGCCATTTCTGCTGATGCGAGTGAAAGCTGAGGTAG
pet28b-ALC1_1-880:R260M_R	CTACCTCAGCTTTCACTCGCATCAGCAGAAATGGCTGCAAG
pet28b-ALC1_1-880:R319Q_F	GAACATTTTGTCCCAGCTTCAAAAGTGTGTGGATCACCC
pet28b-ALC1_1-880:R319Q_R	GGGTGATCCACACACTTTTGAAGCTGGGACAAAATGTTC
pet28b-ALC1_1-880:R457H_F	GCTGCCAGGGCTCATCACATTGGCCAAAACAAGTCTG
pet28b-ALC1_1-880:R457H_R	CAGACTTGTTTTGGCCAATGTGATGAGCCCTGGCAGC
pet28b-ALC1_1-880:D381A_F	CCAGATGTTGGATATTCTCCAAGCCTATATGGATTACAGAGGCTACAG
pet28b-ALC1_1-880:D381A_R	CTGTAGCCTCTGTAATCCATATAGGCTTGGAGAATATCCAACATCTGG
pet28b-ALC1_1-880:W852C_F	CACGAAAGGTTTTAACTGCTATGGTACTGAGCGAC
pet28b-ALC1_1-880:W852C_R	GTCGCTCAGTACCATAGCAGTTAAAACCTTTCGTG
pet28b-ALC1_1-880:F	GGGTACCACGCGTGGATCCGGAGGTAGTACAATGGAGCGCGCGGGCGCTA
pet28b-ALC1_1-880-avi:R2	TTCTGGGCCTCGAAGATATCGTTCAGGCCGCCGCTGCTAGACTTGCTTCTAGGAAAATA
pet28b-ALC1_1-880-avi:R1	TGGTGCTCGAGTGCGGCCGCATTACTCGTGCCACTCGATCTTCTGGGCCTCGAAGATAT
pVRC8400-scFv_1-257:F	CCTTTTTCTAGTAGCAACTGCAACCGGTGTACATTCCCAGGTACAGCTGCAGCAGTC
pVRC8400-scFv_1-257:R	TTAGTGATGGTGATGGTGATGTAGAATTCCGGATAGGACGG
pet28b-HSPARP1_1-1015:F	GGGTACCACGCGTGGATCCATGGCGGAGTCTTCGGAT
pet28b-HSPARP1_1-1015:R	TGGTGCTCGAGTGCGGCCGCATTACCACAGGGAGGTCTTAA
pet15b-H2A_1-130:E56K/E61K-F	ATGTCAGGAAGAGGCAAACAAG
pet15b-H2A_1-130:E56K/E61K-1R	GCGAGTCTTCTTGTTATCACGGGCCGCATTCCCGGCCAATTCCAAAATCTTAGCGGTCAGATACTTCAACACTGCAGCCAGATAG
pet15b-H2A_1-130:D90R/E92K-2F	GTGATAACAAGAAGACTCGCATTATCCCCAGACACCTGCAGCTCGCTGTGCGCAACAAGGAGAAACTGAACAAACTGCTCGGAAG
pet15b-H2A_1-130:D90R/E92K-R	TCACTTGCTCTTGGCCGAC

Supplementary References

1 Li, M. *et al.* Mechanism of DNA translocation underlying chromatin remodelling by Snf2. Nature 567, 409-413 (2019).

2 Pettersen, E. F. *et al.* UCSF Chimera--a visualization system for exploratory research and analysis. J. Comput. Chem. 25, 1605-1612 (2004).

3 Jankevicius, G., Ariza, A., Ahel, M. & Ahel, I. The Toxin-Antitoxin System DarTG Catalyzes Reversible ADP-Ribosylation of DNA. Mol. Cell 64, 1109-1116 (2016).

4 Timinszky, G. *et al.* A macrodomain-containing histone rearranges chromatin upon sensing PARP1 activation. Nat. Struct. Mol. Biol. 16, 923-929 (2009).

5 Madeira, F. *et al.* The EMBL-EBI search and sequence analysis tools APIs in 2019. Nucleic Acids Res. 47, W636-W641 (2019).
