# Supplementary material for: Structural basis of ALC1/CHD1L autoinhibition and the mechanism of activation by the nucleosome
Source: Nat Commun. 2021 Jul 1;12:4057. doi: 10.1038/s41467-021-24320-4 (PMC8249414; doi:10.1038/s41467-021-24320-4)
Supplement: Supplementary file 4 — Source Data [file 41467_2021_24320_MOESM4_ESM.zip › source data/scfv_VH-identify information.pdf]

IGBLAST» JOB ID: jpkJKAqWJ6IMmTvDIb5dxii3TNU
Formatting Results

Database: imgt.Homo_sapiens.V.f.orf.p; imgt.Homo_sapiens.D.f.orf;
imgt.Homo_sapiens.J.f.orf
622 sequences; 165,139 total letters

Query=
Length=390

Sequences producing significant alignments:	Score (Bits)	E Value
IGHV6-1*01germline gene	422	3e-120
IGHV6-1*02germline gene	419	3e-119
IGHV6-1*03germline gene	419	3e-119
IGHD1-1*01germline gene	12.2	39
IGHD1-20*01germline gene	12.2	39
IGHD1-26*01germline gene	12.2	39
IGHJ3*02germline gene	81.4	1e-19
IGHJ3*01germline gene	75.7	7e-18
IGHJ4*03germline gene	50.7	2e-10

Domain classification requested: imgt

V-(D)-J rearrangement summary for query sequence (multiple equivalent top matches, if present, are separated by a comma):

Top V gene match	Top D gene match	Top J gene match	Chain type	stop codon	V-J frame	Productive	Strand	V frame shift
IGHV6-1*01	IGHD1-1*01,IGHD1-20*01,IGHD1-26*01	IGHJ3*02	VH	No	In-frame	Yes	+	No

V-(D)-J junction details based on top germline gene matches:

V region end	V-D junction*	D region	D-J junction*	J region start
AAGAG	CTACTAGGCCTACTG	GGAACG	GTC	TTGAT

*: Overlapping nucleotides may exist at V-D-J junction (i.e, nucleotides that could be assigned to either rearranging gene). Such nucleotides are indicated inside a parenthesis (i.e., (TACAT)) but are not included under the V, D or J gene itself.

Sub-region sequence details:

	Nucleotide sequence	Translation	Start	End
CDR3	GCAAGAGCTACTAGGCCTACTGGGAACGGTCTTGATATC	ARATRPTGNGLDI	304	342

Alignment summary between query and top germline V gene hit:

	from	to	length	matches	mismatches	gaps	identity(%)
FR1-IMGT	7	81	75	73	2	0	97.3
CDR1-IMGT	82	111	30	25	5	0	83.3
FR2-IMGT	112	162	51	49	2	0	96.1
CDR2-IMGT	163	189	27	26	1	0	96.3
FR3-IMGT	190	303	114	107	7	0	93.9
CDR3-IMGT (germline)	304	310	7	7	0	0	100
Total			304	287	17	0	94.4

Alignments

```
                                                <-------------------------------FR1-IMGT-------------------------------><----------CDR1
                                                 Q  V  Q  L  Q  Q  S  G  P  G  L  V  R  P  S  Q  T  L  S  L  T  C  A  I  S  G  D  S  V  S
                           Query_1      7       CAGGTACAGCTGCAGCAGTCAGGCCCAGGACTGGTGAGGCCCTCGCAGACCCTCTCACTCACCTGTGCCATCTCCGGGGACAGTGTCTCT  96
V  94.4% (287/304)  IGHV6-1*01   1       .......................T.............A....................................................  90
                                                 Q  V  Q  L  Q  Q  S  G  P  G  L  V  K  P  S  Q  T  L  S  L  T  C  A  I  S  G  D  S  V  S
V  94.1% (286/304)  IGHV6-1*02   1       .......................T..G..........A....................................................  90
V  94.1% (286/304)  IGHV6-1*03   1       .......................T.............A....................................................  90

                                                -IMGT--------><------------------FR2-IMGT------------------><--------CDR2-IMGT------
                                                 D  N  D  A  A  W  N  W  V  R  Q  S  P  S  R  G  L  E  W  L  G  R  T  Y  Y  R  S  K  W  S
                           Query_1      97      GACAACGATGCTGCCTGGAACTGGGTCAGGCAGTCCCCATCGCGAGGCCTTGAGTGGCTGGGAAGGACATACTACAGGTCCAAGTGGTCT  186
V  94.4% (287/304)  IGHV6-1*01   91      AG....AG......T.........A.................A.............................................A.  180
                                                 S  N  S  A  A  W  N  W  I  R  Q  S  P  S  R  G  L  E  W  L  G  R  T  Y  Y  R  S  K  W  Y
V  94.1% (286/304)  IGHV6-1*02   91      AG....AG......T.........A.................A.............................................A.  180
V  94.1% (286/304)  IGHV6-1*03   91      AG....AG......T.........A.................A.............................................A.  180

                                                --><--------------------------------------------------FR3-IMGT------------------------
                                                 N  E  Y  A  V  F  V  Q  S  R  I  T  I  N  P  D  T  S  K  N  Q  F  S  L  H  L  N  S  V  T
                           Query_1      187     AATGAATATGCAGTATTTGTGCAAAGTCGAATAACCATCAACCCAGACACATCCAAGAATCAGTTCTCCCTGCACTTGAACTCTGTGACT  276
V  94.4% (287/304)  IGHV6-1*01   181     .....T..........C....A.....................................C..............GC..............  270
                                                 N  D  Y  A  V  S  V  K  S  R  I  T  I  N  P  D  T  S  K  N  Q  F  S  L  Q  L  N  S  V  T
V  94.1% (286/304)  IGHV6-1*02   181     .....T..........C....A.....................................C..............GC..............  270
V  94.1% (286/304)  IGHV6-1*03   181     .....T..........C....A.....T...............................C..............GC..............  270

                                                -------------------------><-------------CDR3-IMGT-------------><----------FR4-IMGT----
                                                 P  E  D  T  A  V  Y  Y  C  A  R  A  T  R  P  T  G  N  G  L  D  I  W  G  Q  G  T  M  V  T
                           Query_1      277     CCCGAGGACACGGCTGTCTATTACTGTGCAAGAGCTACTAGGCCTACTGGGAACGGTCTTGATATCTGGGGCCAAGGGACAATGGTCACC  366
V  94.4% (287/304)  IGHV6-1*01   271     .................G................--------------------------------------------------------  304
                                                 P  E  D  T  A  V  Y  Y  C  A  R
V  94.1% (286/304)  IGHV6-1*02   271     .................G................--------------------------------------------------------  304
V  94.1% (286/304)  IGHV6-1*03   271     .................G................--------------------------------------------------------  304
D  100.0% (6/6)     IGHD1-1*01   10      -------------------------------------------------......-----------------------------------  15
D  100.0% (6/6)     IGHD1-20*01  10      -------------------------------------------------......-----------------------------------  15
D  100.0% (6/6)     IGHD1-26*01  14      ----------------------------------......--------------------------------------------------  19
J  100.0% (42/42)   IGHJ3*02     9       ----------------------------------------------------------................................  40
J  97.6% (41/42)    IGHJ3*01     9       ----------------------------------------------------------.....G..........................  40
J  91.4% (32/35)    IGHJ4*03     14      -----------------------------------------------------------------...............CC........  38

                                                -------->
                                                 V  S  S
                           Query_1      367     GTCTCTTCAG  376
J  100.0% (42/42)   IGHJ3*02     41      ..........  50
J  97.6% (41/42)    IGHJ3*01     41      ..........  50
J  91.4% (32/35)    IGHJ4*03     39      .....C....  48
```

```
Lambda      K        H
    1.10    0.333    0.549

Gapped
Lambda      K        H
    1.08    0.280    0.540

Effective search space used: 53328754
```

```
Total queries = 1
Total identifiable CDR3 = 1
Total unique clonotypes = 1


  Database: imgt.Homo_sapiens.V.f.orf.p
    Posted date:  Apr 2, 2021  12:46 PM
  Number of letters in database: 162,885
  Number of sequences in database:  556

  Database: imgt.Homo_sapiens.D.f.orf
    Posted date:  May 17, 2012  12:56 PM
  Number of letters in database: 828
  Number of sequences in database:  34

  Database: imgt.Homo_sapiens.J.f.orf
    Posted date:  May 17, 2012  12:56 PM
  Number of letters in database: 1,426
  Number of sequences in database:  32


Matrix: blastn matrix 1 -1
Gap Penalties: Existence: 4, Extension: 1
```

- National Library Of Medicine
- National Institutes Of Health
- U.S. Department of Health & Human Services
- USA.gov

NCBI

National Center for Biotechnology Information, U.S. National Library of Medicine 8600 Rockville Pike, Bethesda MD, 20894 USA
Policies and Guidelines | Contact
