# Supplementary material for: Structural basis of ALC1/CHD1L autoinhibition and the mechanism of activation by the nucleosome
Source: Nat Commun. 2021 Jul 1;12:4057. doi: 10.1038/s41467-021-24320-4 (PMC8249414; doi:10.1038/s41467-021-24320-4)
Supplement: Supplementary file 4 — Source Data [file 41467_2021_24320_MOESM4_ESM.zip › source data/scfv_VL-identify information.pdf]

IGBLAST» JOB ID: uKZdSOBKWWZqxm7wN-tNljbiSIcp
Formatting Results

```
Database: imgt.Homo_sapiens.V.f.orf.p; imgt.Homo_sapiens.D.f.orf;
imgt.Homo_sapiens.J.f.orf
           622 sequences; 165,139 total letters



Query=
Length=336
                                                                                                           Score     E
Sequences producing significant alignments:                                                               (Bits)  Value

IGLV3-21*02germline gene                                                                                   441      7e-126
IGLV3-21*03germline gene                                                                                   438      6e-125
IGLV3-21*04germline gene                                                                                   428      4e-122
IGLJ2*01germline gene                                                                                      64.1     2e-14
IGLJ3*01germline gene                                                                                      64.1     2e-14
IGLJ3*02germline gene                                                                                      54.5     1e-11


Domain classification requested: imgt
```

V-(D)-J rearrangement summary for query sequence (multiple equivalent top matches, if present, are separated by a comma):

| Top V gene match | Top J gene match | Chain type | stop codon | V-J frame | Productive | Strand | V frame shift |
|---|---|---|---|---|---|---|---|
| IGLV3-21*02 | IGLJ2*01,IGLJ3*01 | VL | No | In-frame | Yes | + | No |

V-(D)-J junction details based on top germline gene matches:

| V region end | V-J junction* | J region start |
|---|---|---|
| ATCAT | | GTGGT |

*: Overlapping nucleotides may exist at V-D-J junction (i.e, nucleotides that could be assigned to either rearranging gene). Such nucleotides are indicated inside a parenthesis (i.e., (TACAT)) but are not included under the V, D or J gene itself.

Sub-region sequence details:

| | Nucleotide sequence | Translation | Start | End |
|---|---|---|---|---|
| CDR3 | CAGGTGTGGGATAGTAGTAGTGATCATGTGGTA | QVWDSSSDHVV | 262 | 294 |

Alignment summary between query and top germline V gene hit:

| | from | to | length | matches | mismatches | gaps | identity(%) |
|---|---|---|---|---|---|---|---|
| FR1-IMGT | 1 | 75 | 75 | 74 | 1 | 0 | 98.7 |
| CDR1-IMGT | 76 | 93 | 18 | 17 | 1 | 0 | 94.4 |
| FR2-IMGT | 94 | 144 | 51 | 51 | 0 | 0 | 100 |
| CDR2-IMGT | 145 | 153 | 9 | 9 | 0 | 0 | 100 |
| FR3-IMGT | 154 | 261 | 108 | 107 | 1 | 0 | 99.1 |
| CDR3-IMGT (germline) | 262 | 288 | 27 | 27 | 0 | 0 | 100 |
| Total | | | 288 | 285 | 3 | 0 | 99 |

Alignments

```
                                       <--------------------------------FR1-IMGT--------------------------------><----CDR1-IMGT-
                                        S  Y  V  L  T  Q  P  P  S  V  S  V  A  P  G  Q  T  A  R  I  T  C  G  G  N  N  I  G  S  K
                   Query_1      1      TCCTATGTGCTGACACAGCCACCCTCGGTGTCAGTGGCCCCAGGACAGACGGCCAGGATTACCTGTGGGGGAAACAACATTGGCAGTAAA  90
V  99.0% (285/288) IGLV3-21*02  1      ..............T....................................................................A......  90
                                        S  Y  V  L  T  Q  P  P  S  V  S  V  A  P  G  Q  T  A  R  I  T  C  G  G  N  N  I  G  S  K
V  98.6% (284/288) IGLV3-21*03  1      ..............T..............................A.....................................A......  90
V  97.6% (281/288) IGLV3-21*04  1      ..............T...........A..................A.....................................A......  90

                                       -->          <--------------------FR2-IMGT--------------------><CDR2-IM><-------------------------
                                        S  V  H  W  Y  Q  Q  K  P  G  Q  A  P  V  L  V  V  Y  D  D  S  D  R  P  S  G  I  P  E  R
                   Query_1      91     AGTGTGCACTGGTACCAGCAGAAGCCAGGCCAGGCCCCTGTGCTGGTCGTCTATGATGATAGCGACCGGCCCTCAGGGATCCCTGAGCGA  180
V  99.0% (285/288) IGLV3-21*02  91     ..........................................................................................  180
                                        S  V  H  W  Y  Q  Q  K  P  G  Q  A  P  V  L  V  V  Y  D  D  S  D  R  P  S  G  I  P  E  R
V  98.6% (284/288) IGLV3-21*03  91     ..........................................................................................  180
V  97.6% (281/288) IGLV3-21*04  91     ................................................A.....T...................................  180

                                       ----------------------FR3-IMGT------------------------------------------------><--------
                                        F  S  G  S  N  S  G  N  T  A  A  L  T  I  S  R  V  E  A  G  D  E  A  D  Y  Y  C  Q  V  W
                   Query_1      181    TTCTCTGGCTCCAACTCTGGGAACACGGCCGCCCTGACCATCAGCAGGGTCGAAGCCGGGGATGAGGCCGACTATTACTGTCAGGTGTGG  270
V  99.0% (285/288) IGLV3-21*02  181    ..............................A...........................................................  270
                                        F  S  G  S  N  S  G  N  T  A  T  L  T  I  S  R  V  E  A  G  D  E  A  D  Y  Y  C  Q  V  W
V  98.6% (284/288) IGLV3-21*03  181    ..............................A...........................................................  270
V  97.6% (281/288) IGLV3-21*04  181    ..............................A...........................................................  270

                                       ---CDR3-IMGT----------><----------FR4-IMGT---------->
                                        D  S  S  S  D  H  V  V  F  G  G  G  T  K  L  T  V  L
                   Query_1      271    GATAGTAGTAGTGATCATGTGGTATTCGGCGGAGGGACCAAGCTCACCGTCCTA  324
V  99.0% (285/288) IGLV3-21*02  271    ..................------------------------------------  288
                                        D  S  S  S  D  H
V  98.6% (284/288) IGLV3-21*03  271    ..................------------------------------------  288
V  97.6% (281/288) IGLV3-21*04  271    ..................------------------------------------  288
J  97.2% (35/36)   IGLJ2*01     2      ------------------..........................G.........  37
J  97.2% (35/36)   IGLJ3*01     2      ------------------..........................G.........  37
J  94.1% (32/34)   IGLJ3*02     4      --------------------...G....................G.........  37
```

```
Lambda      K        H
    1.10    0.333    0.549

Gapped
Lambda      K        H
    1.08    0.280    0.540

Effective search space used: 45373636
```

---

```
Total queries = 1
Total identifiable CDR3 = 1
Total unique clonotypes = 1


  Database: imgt.Homo_sapiens.V.f.orf.p
    Posted date:  Apr 2, 2021  12:46 PM
  Number of letters in database: 162,885
  Number of sequences in database:  556

  Database: imgt.Homo_sapiens.D.f.orf
    Posted date:  May 17, 2012  12:56 PM
  Number of letters in database: 828
  Number of sequences in database:  34

  Database: imgt.Homo_sapiens.J.f.orf
    Posted date:  May 17, 2012  12:56 PM
  Number of letters in database: 1,426
  Number of sequences in database:  32


Matrix: blastn matrix 1 -1
Gap Penalties: Existence: 4, Extension: 1
```


BLAST is a registered trademark of the National Library of Medicine

Support center Mailing list YouTube

- NATIONAL LIBRARY OF MEDICINE National Library Of Medicine
- NIH National Institutes Of Health
- U.S. Department of Health & Human Services

- USA.gov

## NCBI

National Center for Biotechnology Information, U.S. National Library of Medicine 8600 Rockville Pike, Bethesda MD, 20894 USA
Policies and Guidelines | Contact
