# Supplementary material for: Structural basis of ALC1/CHD1L autoinhibition and the mechanism of activation by the nucleosome
Source: Nat Commun. 2021 Jul 1;12:4057. doi: 10.1038/s41467-021-24320-4 (PMC8249414; doi:10.1038/s41467-021-24320-4)
Supplement: Supplementary file 4 — Source Data [file 41467_2021_24320_MOESM4_ESM.zip › source data/sliding gels for Figs. 3c,4d,5b,5g-h and Suppl. Figs. 8,9.pptx]

## Slide 1
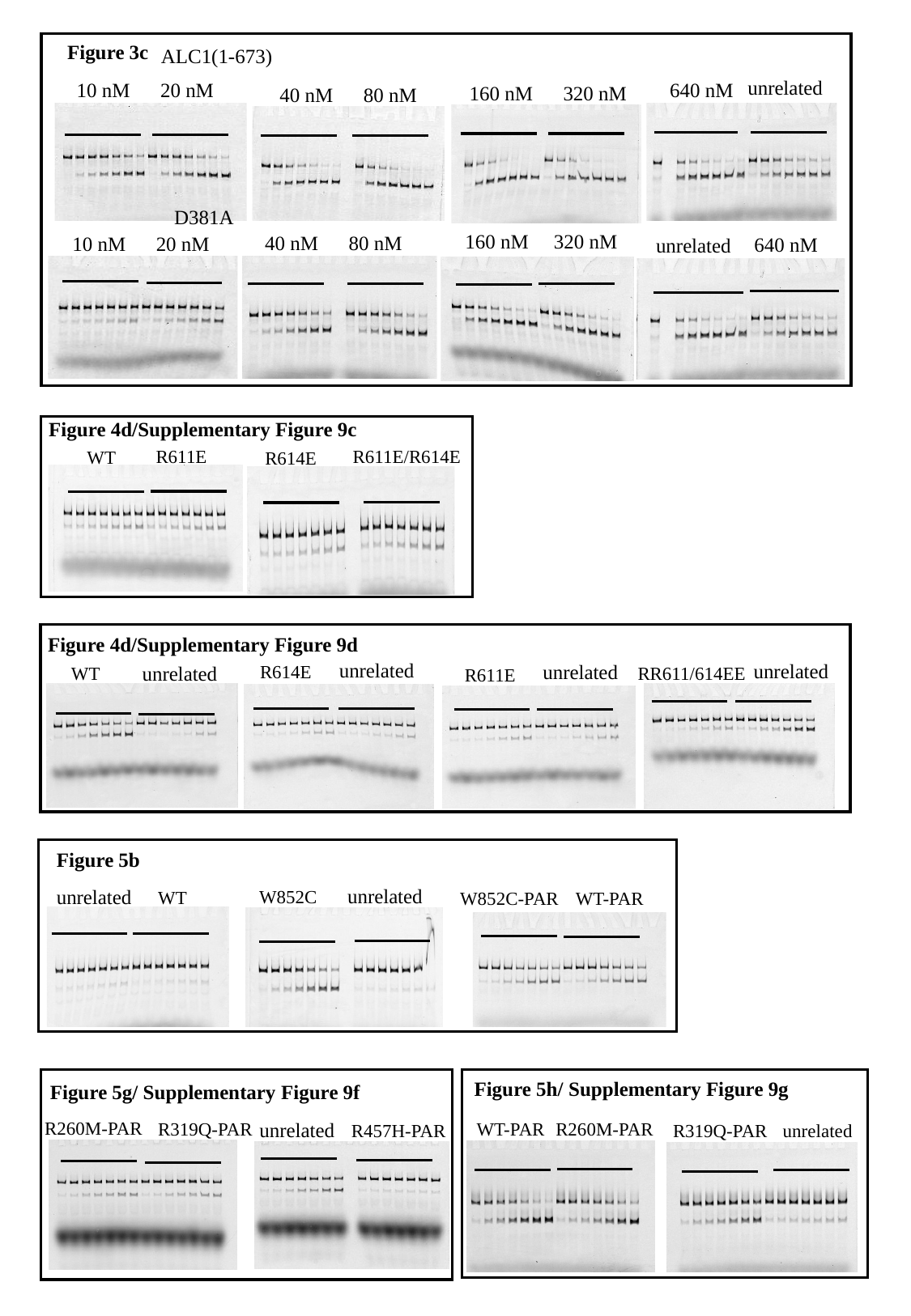

Figure 3c
unrelated
640 nM
10 nM 20 nM
160 nM 320 nM
40 nM 80 nM
160 nM 320 nM
40 nM 80 nM
10 nM 20 nM
640 nM
unrelated
ALC1(1-673)
D381A
Figure 4d/Supplementary Figure 9c
R611E/R614E
R614E
R611E
WT
unrelated
R614E
unrelated
RR611/614EE
unrelated
R611E
unrelated
WT
Figure 4d/Supplementary Figure 9d
unrelated
W852C
WT
W852C-PAR
WT-PAR
unrelated
Figure 5b
R260M-PAR
R319Q-PAR
unrelated
R457H-PAR
Figure 5g/ Supplementary Figure 9f
R260M-PAR
WT-PAR
R319Q-PAR
unrelated
Figure 5h/ Supplementary Figure 9g

## Slide 2
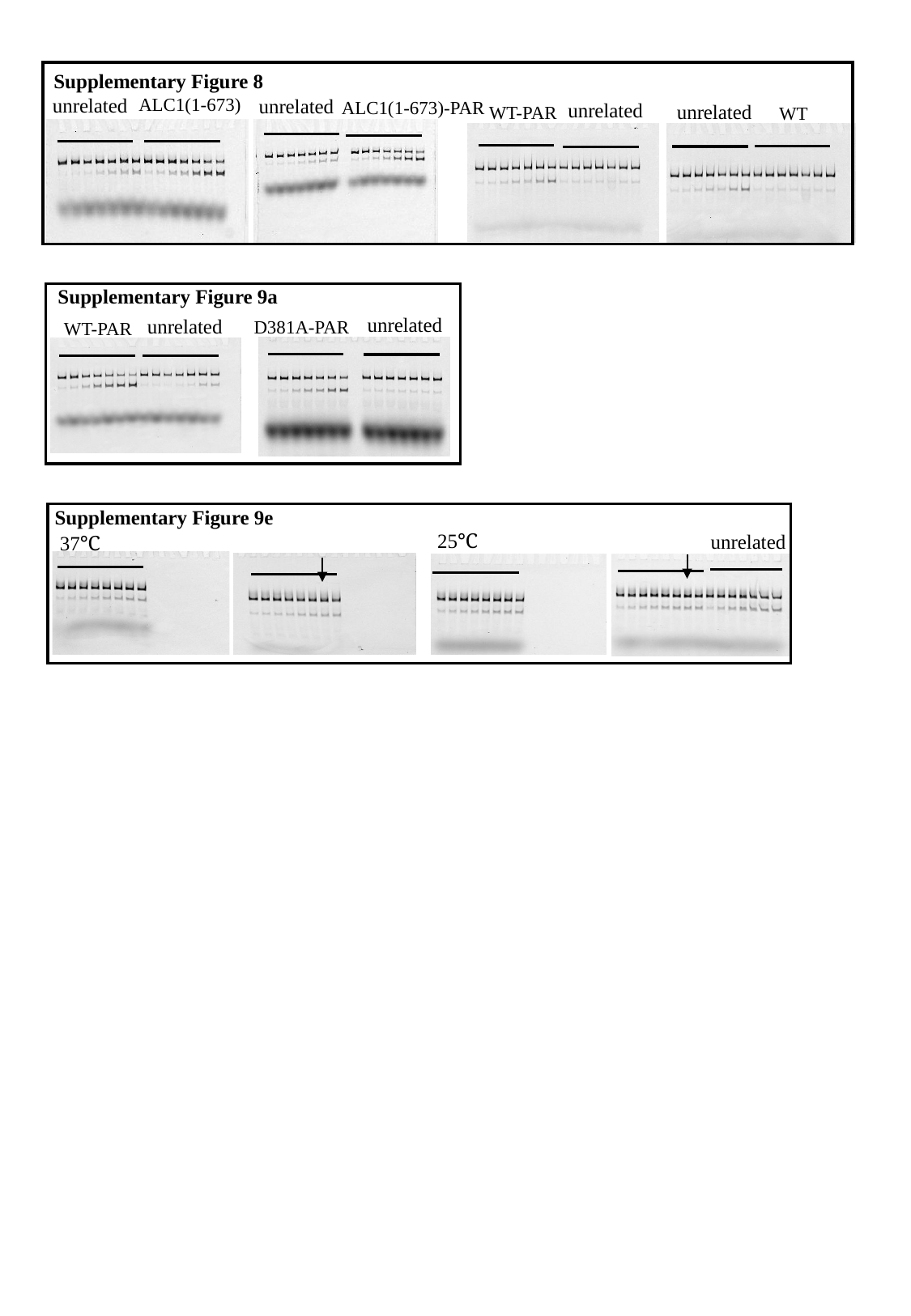

Supplementary Figure 8
unrelated
ALC1(1-673)
unrelated
ALC1(1-673)-PAR
unrelated
WT-PAR
unrelated
WT
Supplementary Figure 9a
unrelated
D381A-PAR
unrelated
WT-PAR
Supplementary Figure 9e
unrelated
25℃
37℃
